# Relationship between psychiatric disorders and loss weight among patients underwent metabolic and bariatric surgery: A reassessment observational study after nine years

**DOI:** 10.1016/j.clinsp.2024.100517

**Published:** 2024-10-20

**Authors:** Leorides Severo Duarte-Guerra, Julia Faria Villares, Marco Aurélio Santo, Francisco Lotufo-Neto, Yuan-Pang Wang

**Affiliations:** a Department of Psychiatry, Faculdade de Medicina da Universidade de São Paulo (FMUSP), São Paulo, SP, Brazil; b Centro Universitário Fundação Santo André, Santo André, SP, Brazil; c Department of Digestive Surgery, Faculdade de Medicina da Universidade de São Paulo (FMUSP), São Paulo, SP, Brazil

**Keywords:** Bariatric surgery, Obesity, Psychiatric disorders, Weight loss

## Abstract

**Objective:**

To evaluate the evolution of Psychiatric Disorders (PD) and weight loss at different postoperative intervals up to 9 years after Metabolic and Bariatric Surgery (MBS).

**Methods:**

This is a nine-year longitudinal study conducted at a single university-based bariatric center in Brazil. The Generalized Estimating Equation (GEE) and multiple linear regression models were used to evaluate the proportion of lifetime PD according to TPS, and its relationship with weight. The Structured Clinical Interview for DSM-5 assessed the PD and the percentage of Excess Lost Weight (%EWL) for weight changes. The study included 142 participants who had undergone MBS, mostly women (82 %), mean age of 52 years.

**Results:**

Depression and anxiety disorders were the most common PD. Regardless of the time elapsed since MBS, there was an increase of 32 % in lifetime mood disorders, bipolar disorders, and eating disorders. The average EWL for Time Post-Surgery (TPS) ≤ 24 months was 69 % and remained consistent until 72 months; after was 57 % and 58 % for ≤ 72 and 96 months, respectively. There was not a significant association between %EWL and PD when controlling for sex, age, and time post-surgery.

**Conclusion:**

Psychiatric disorders can be associated and remain prevalent, regardless of time post-surgery; however, this does not significantly impact weight losss maintenance. It is recommended that individuals who have undergone MBS be monitored and supported over an extended period to address psychiatric comorbidities.

## Highlights

• Psychiatric disorders can be associated with bariatric surgery, regardless of elapsed time.
• Depression and anxiety were the most common conditions among operated patients.
• Lifetime mood disorders, bipolar disorder, and eating disorders increase significantly in post-bariatric surgery.
• Psychiatric disorders were not associated with recurrent weight gain over time.

## Introduction

Obesity is among the most predominant metabolic diseases, accounting for about two-thirds of the global burden of disease^1^ and severe obesity has increased worldwide, from 4.7 % to 9.2 %.^2^ Various surgical procedures are available to address obesity^3^ and Metabolic and Bariatric Surgery (MBS) has been shown to be effective for significant weight loss, management of comorbidities, enhancement of quality of life, and increased life expectancy in patients with severe obesity.^3^ However, Psychiatric Disorders (PDs) and their associated complications play a significant role in both the pre- and post-surgical periods of obesity.^4^

In the North American adult population, obesity is associated with a 25 % increase in the chances of the individual having a mood or anxiety disorder.^5^ The literature indicates a relationship between PD and weight loss after bariatric surgery, regardless of the type of procedure.^6^ Although some of these procedures have been used for decades, the long-term role played by surgical intervention in patients’ mental health remains under-explored. Most MBS research has focused on mental health outcomes within the first year of surgery. However, while mental health indicators usually improve in the first or second year after MBS, this improvement tends to decline by the third year. After ten years of follow-up, a subsequent decline in mental health has been observed.^4^^,^^7^^,^^8^ The literature revealed that mental health complications, including suicidal ideation and substance abuse, are more prevalent among bariatric patients than the general population.^9^ Depressive disorders decreased after MBS but regressed to baseline levels after three years post-surgery.^9^^,^^10^ Furthermore, some studies have associated depressive and anxiety disorders with patterns of weight loss and regain following MBS.^6^

Lifetime PD, especially mood, anxiety disorders, and binge eating behaviors, is linked to a reduced weight loss trajectory over a 50-month period following MBS. This effect is more pronounced in those with two or more PDs.^10^, ^11^, ^12^ It is possible that individuals who undergo MBS may be more susceptible to developing PDs or exacerbating pre-existing PDs which may have been masked by overeating.^13^ In addition, obesity has been associated with neuropsychiatric problems, indicating a likely connection to its development. Recent studies propose a bidirectional pathophysiological link between these conditions, suggesting a possible “psychopathology-to-obesity” pathway.^14^ In other words, it implies that certain psychological or psychiatric conditions may influence behaviors, habits, or physiological processes that can lead to obesity. However, the mental health outcomes of patients who have undergone MBS have not been thoroughly investigated at different postoperative intervals.

A seven-year longitudinal study has revealed an inconsistent relationship between PDs, weight loss, and Time Post-Surgery (TPS).^6^ The literature indicates that psychiatric diagnoses can show significant individual variability in symptom severity and expression.^15^ However, the progression of PDs at various time points following MBS has not been extensively investigated. Furthermore, there is a notable scarcity of literature on the long-term progression of mental disorders in patients undergoing surgical procedures, and such studies as there are often focused on analyzing a single disorder or comparing two disorders. The present study addresses this gap in the literature and proposes a hypothesis regarding the potential bidirectional relationship between the frequency of PDs and weight loss patterns across different post-surgery intervals. In addition, the authors offer a comprehensive overview of mental disorders using a standardized instrument with the same population over an extended period. PD diagnosis was determined using the Structured Clinical Interview for DSM-5 (SCID-5).^16^

In a study conducted nine years ago, it was found that 81 % of individuals in the MBS preoperative program met the criteria for at least one PD in their lifetime.^17^ Accordingly, the principal objective of the present study was to reassess the same sample in order to estimate and compare the frequency of PDs among patients at different postoperative intervals up to nine years after MBS. Furthermore, the authors investigated the potential correlation between these PDs and weight changes over time, focusing exclusively on patients who had undergone MBS. This approach allows for a more comprehensive analysis of the progression of PD in the postoperative period.

## Methods

### Design, setting, and participants

**Fig. 1:**
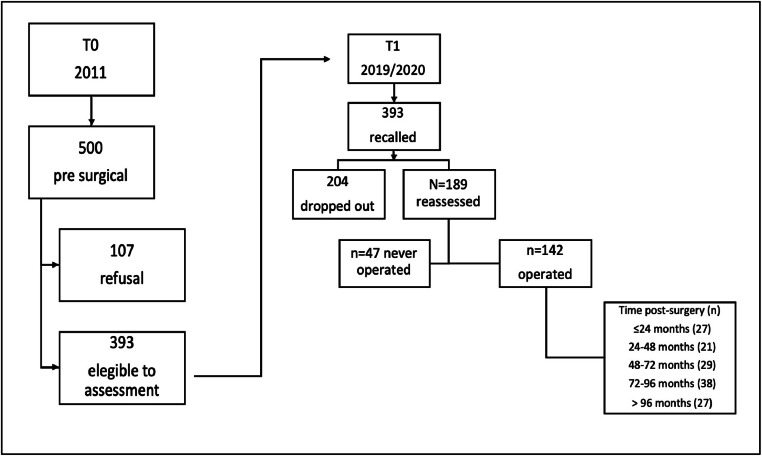
Flowchart of the sampling (T_0_ 2011 and T_1_ 2019/2020).

This study is an extension of the cross-sectional assessment conducted in 2011.^17^ The original sample comprised 393 patients who were recruited from a university hospital-based bariatric center waiting list. The 2011 study revealed that 57.8 % of patients met the criteria for any Personality Disorder (PD) within the past month, while 80.9 % had met the criteria at some point in their lifetime.^17^ A total of 393 patients who participated in the initial phase of the study (February 2019 to March 2020) were recruited, irrespective of whether they had undergone MBS or the specific type of procedure performed. The study sample was ultimately limited to those who had undergone the surgical procedure, thereby ensuring the inclusion of individuals with pre-existing psychiatric conditions prior to surgery. A total of 204 participants dropped out of the study (six died; three relocated to another city, four refused to continue participating, 26 were unable to participate due to the Coronavirus Disease 2019 (COVID-19) lockdown, and 165 could not be located for reassessment), leaving 189 patients for re-evaluation. Of these, 47 had not undergone MBS. The remaining 142 patients had already undergone MBS. The present study sample consisted solely of the 142 participants who underwent the surgical procedure. The data obtained in 2011 was designated as T_0_, while the data obtained in 2019/2020 was designated as T_1_. A comparison of the sociodemographic characteristics of participants who were followed up in 2019/2020 with those who were revealed no significant differences. All manuscripts were prepared following the Strengthening the Reporting of Observational Studies in Epidemiology (STROBE) guidelines (Supplementary Material – Table S1, and Fig. 1).

Table 1 shows the frequency and descriptive statistics of the demographic and clinical characteristics of the participants at T_1_ (2019/2020). The sample predominantly consisted of women (82 %) with a mean age of 52 years (SD = 11.3, range: 29‒86 years). Half of the participants were married (51 %), and the average schooling duration was ten years (SD = 3.8). Most participants were employed (59 %) and 9 % reported current tobacco use. Of the 142 individuals, 91 % (*n* = 129) underwent Roux-en-Y Gastric Bypass (RYGB), while the remaining 9 % (*n* = 13) underwent the sleeve gastrectomy procedure. Due to the limited number of sleeve procedures, the authors did not consider the difference between the procedures.

**Table 1:** Socio-demographic characteristics of patients with severe obesity (2019‒2020), who have undergone bariatric surgery.

	Bariatric Surgery, *n* = 142 (%)
**Sex (n, %)**	
Women	117 (82)
Men	25 (18)
Age means (SD)	52 (11)
**Marital Status (n, %)**	
Married	73 (51)
Widow	16 (11)
Separated	29 (20)
Single	24 (17)
**Education (n, %)**	
1‒8 years	48 (34)
9‒12 years	55 (39)
>12 years	39 (27)
**Employed (n, %)**	
Yes	84 (59)
Not	58 (41)
**Tobacco use (n, %)**	
Current smoker	13 (9)
Ex-smoker	52 (37)
**BMI (Kg/m^2^) mean (SD)**	34 (6)
**GAF (0‒100) mean (SD)**	79 (13)

SD, Standard Deviation; BMI, Body Mass Index; GAF, Global Assessment of Function.

### Assessment tool and TPS

The Structured Clinical Interview for DSM-5 (SCID-5),^16^ used to diagnose PD in this study, is the standard instrument used for diagnosing the PDs listed in DSM-5.^18^ After screening for the presence of core psychopathology, the interviewer should determine if the respondent meets the criteria for affective disorders (sections A, D), psychotic disorders (C, D), substance use disorders (SUD) (E), anxiety disorders (F), somatoform disorders (G), eating disorders (H), and adjustment disorders (I). The diagnostic strategy adopted by SCID-5 is top-down, i.e., the presence of symptoms should be checked against the criteria for the suspected psychiatric diagnosis. The section on psychotic disorders and somatoform disorders was omitted to shorten the time taken to apply the instrument.

Nine researchers (eight psychologists and one psychiatrist) acquainted with clinical obesity were trained in the administration of SCID-5. The course included a 60hr calibration practice. Inter-rater agreement was assessed within an arbitrary triplet of researchers for the first 18 patients. The Fleiss’ kappa coefficient^19^ for lifetime psychiatric disorders was κ = 72. At the end of the encounter, the level of Global Assessment Functioning (GAF) was scored by the interviewers, based on the participant's best role functioning in the past year. From February 2019 to March 2020, eligible patients were randomly assigned to be face-to-face assessed using SCID-5. The average duration of the interview ranged from 60 to 90 min. Participants were divided into five groups based on TPS intervals (≤ 24 to > 96 months) (Fig. 1). These intervals are linked to weight changes after surgery, followed by stabilization within two to three years.^20^

### Outcomes

#### Psychiatric disorders

Some diagnostic criteria changed between DSM-IV and DSM-5 in 2013. A limited number of studies initially assessed conditions based on DSM-IV and later reevaluated them with DSM-5. A recent study found that DSM-5 criteria generally yielded comorbidity rates that were similar to or lower than DSM-IV.^21^ In this study, the authors assessed current (last month) and lifetime psychiatric disorders from 2011 onwards, as earlier data had already been presented (T0/2011).^17^ The evaluation at T1 (2019‒2020), included the following categories: bipolar disorder (BD-I, BD-II, BD Not Otherwise Specified[NOS]), mood disorder (major depressive disorder, persistent depressive disorder), substance use disorder (alcohol and drugs), eating disorder (anorexia nervosa, bulimia nervosa, binge eating), anxiety disorders (panic disorder, social phobia, specific phobia, post-traumatic stress disorder; obsessive-compulsive disorder, anxiety NOS), and any lifetime disorders. It is important to note that Loss of Control (LOC) was the qualifying criterion for Binge Eating Disorder (BED). Although surgical intervention can significantly reduce food intake, individuals who have undergone MBS may still experience Subjective Binge Eating Episodes (SBEs), which involve modest consumption (perceived as large by the individual) and are associated with LOC.^22^^,^^23^

#### Percentage of excess weight loss (%EWL)

A common measure of the percentage of excess Weight Loss (%EWL) was used.^24^ Successful weight loss is typically defined as %EWL > 50 %, while failed weight loss is defined as %EWL < 30 %. Participants self-reported their current weight, pre-operative weight (the weight on the Day Of Surgery [DOS]), and nadir weight (the lowest weight after MBS). A positive %EWL indicates weight loss (current weight < DOS weight), while a negative %EWL indicates weight gain (current weight > DOS weight).

### Ethics and procedures

All participants signed an informed consent before participating in the study. The ethics committee of the University of São Paulo Medical School approved the study (CAPPesq-Protocol 0228/11).

### Statistical analysis

Demographic variables were analyzed using descriptive statistics, including frequencies (%), means, and Standard Deviations (SDs), for both current and lifetime DSM-5 diagnoses. The proportion of lifetime PD according to TPS, was estimated using the Generalized Estimating Equation (GEE).^25^ The correlation structure used for all disorders was compound symmetry, which assumes equal correlation among all patient measures. The models were fitted considering the TPS and evaluation (T_0_ or T_1_) as independent variables, along with the interaction between them. The results were interpreted as Prevalence Ratios (PR).^26^

Multiple linear regression models were used to evaluate the relationship between the variation in weight (%EWL) and the presence of PD at T_0_ and/or T_1_ (PD-T_0_/T_1_). Separate models were built for each disorder. %EWL was the dependent variable, while gender (female), age (average age of 52 years), TPS (≤ 24-months), and PD-T_0_/T_1_ (no disorders) were the independent variables (the reference category). The regression analysis excluded current disorders due to insufficient model adjustments. The low incidence of current disorders may have been influenced by medication use or remission during the interview. Therefore, the analysis focused only on lifetime disorders. The results were analyzed using R version 4.0.2 and expressed as unstandardized coefficients (β) with Standard Errors (SE). Significance testing was conducted at a two-tailed 5 % level.

## Results

Table 2 shows the frequencies (%) of current and lifetime PD at two waves: 2011 (T0) and 2019/2020 (T1), respectively, according to the intervals of the TPS. In T1, 27 (15 %) underwent surgery <24-months ago, 21 (11 %) 24‒48 months ago, 29 (15 %) 48‒72 months ago, 38 (20 %) 72‒96 months ago and 27 (14 %) >96 months ago.

**Table 2:** Absolute and relative frequencies (%) of current and lifetime psychiatric disorders observed in the evaluations conducted in 2011 (T_0_) and in 2019/2020 (T_1_) *n* = 142.

	≤ 24 months post-surgery^a^, *n* = 27 (15 %)		24‒48 months post-surgery, *n* = 21 (11 %)		48‒72 months post-surgery, *n* = 29 (15 %)		72‒96 months post-surgery, *n* = 38 (20 %)		> 96 months post-surgery, *n* = 27 (14 %)	
	T_0_ (%)	T_1_ (%)	T_0_ (%)	T_1_ (%)	T_0_ (%)	T_1_ (%)	T_0_ (%)	T_1_ (%)	T_0_ (%)	T_1_ (%)
**Any Psychiatric Disorders**										
Current	23 (85)	22 (81)	10 (48)	17 (81)	15 (52)	22 (76)	26 (68)	35 (92)	10 (37)	23 (85)
Lifetime	26(96)	27 (100)	15 (71)	20 (95)	22 (76)	25 (86)	35 (92)	38 (100)	19 (70)	25 (93)
**Any Bipolar Disorders**										
Current	5 (19)	10 (37)	1 (5)	8 (38)	3 (10)	12 (41)	6 (16)	15 (39)	3 (11)	9 (33)
Lifetime	15 (56)	14 (52)	5 (24)	13 (62)	12 (41)	16 (55)	17 (45)	23 (61)	9 (33)	11 (41)
**Any Mood Disorders**										
Current	7 (26)	15 (56)	6 (29)	10 (48)	3 (10)	16 (55)	8 (21)	22 (58)	4 (15)	14 (52)
Lifetime	23 (85)	22 (81)	11 (52)	16 (76)	20 (69)	24 (83)	29 (76)	34 (89)	16 (59)	21 (78)
**Any Substance Disorders**										
Current	0 (0)	2 (7)	0 (0)	5 (24)	0 (0)	5 (17)	0 (0)	7 (18)	2 (7)	5 (19)
Lifetime	4 (15)	4 (15)	4 (19)	8 (38)	6 (21)	7 (24)	4 (11)	10 (26)	6 (22)	5 (19)
**Any Anxiety Disorders**										
Current	19 (70)	12 (44)	10 (48)	14 (67)	14 (48)	16 (55)	21 (55)	22 (58)	8 (30)	15 (56)
Lifetime	20 (74)	14 (52)	11 (52)	15 (71)	15 (52)	18 (62)	23 (61)	24 (63)	12 (44)	17 (63)
**Any Eating Disorders**										
Current	9 (33)	7 (26)	1 (5)	5 (24)	3 (10)	10 (34)	10 (26)	24 (63)	2 (7)	8 (30)
Lifetime	14 (52)	17 (63)	6 (29)	8 (38)	8 (28)	16 (55)	15 (39)	26 (68)	4 (15)	12 (44)
**Number disorders**										
**No Disorder**										
Current	4 (15)	5 (19)	11 (52)	4 (19)	14 (48)	7 (24)	12 (32)	3 (8)	17 (63)	4 (15)
Lifetime	1 (4)	0 (0)	6 (29)	1 (5)	7 (24)	4 (14)	3 (8)	0 (0)	8 (30)	2 (7)
**1 Disorder**										
Current	13 (48)	11 (41)	4 (19)	6 (29)	10 (35)	7 (24)	15 (40)	10 (26)	5 (19)	11 (41)
Lifetime	4 (15)	8 (30)	5 (24)	4 (19)	4 (14)	2 (7)	10 (27)	6 (16)	5 (18)	7 (26)
**2 Disorders**										
Current	5 (19)	7 (26)	5 (24)	5 (24)	2 (7)	4 (14)	6 (16)	9 (24)	3 (11)	4 (15)
Lifetime	6 (22)	9 (33)	2 (10)	4 (19)	4 (14)	5 (17)	7 (18)	8 (21)	7 (30)	7 (30)
**3+ Disorders**										
Current	5 (19)	4 (15)	1 (5)	6 (29)	3 (10)	11 (38)	5 (13)	16 (42)	2 (7)	8 (30)
Lifetime	16 (59)	10 (37)	8 (38)	12 (57)	14 (48)	18 (62)	18 (47)	24 (63)	7 (26)	11 (41)

The prevalence of PD in the current period has the highest percentage observed at T_1_ in the 72‒96 months period (92 %). Anxiety and mood disorders were the most common disorders in all periods. Eating disorders showed the highest percentage at T_1_ in the 72‒96 months period (63 %). In terms of frequency, the highest percentage (42 %) of patients with three or more disorders was in the 72‒96 months post-operation period. In terms of lifetime disorders, mood disorders were the most frequent across all time points. Anxiety and eating disorders were the second most frequent conditions in different TPS intervals. The majority of participants exhibited three or more disorders across all TPS intervals, with the highest percentage (63 %) observed in the 72‒96 months post-operation period (Table 2).

**Fig. 2:**
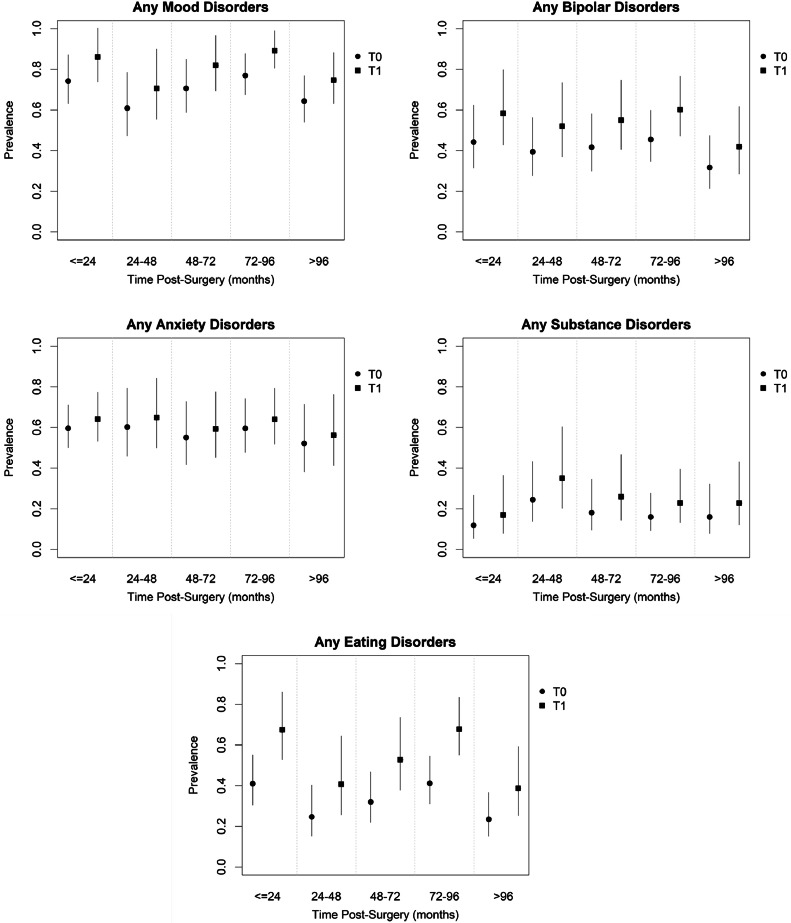
Estimated prevalence and Confidence Interval (CI) 95 % for lifetime psychiatric disorders, according to the time of assessment (T_0_) and the time post-surgery (T_1_)*.*

Fig. 2 shows the estimated Prevalence Ratio (PR) of PD, in T0 and T1, according to TPS, regardless of the time since MBS. There was an increase of 32 % in lifetime mood disorders, bipolar disorders, and eating disorders (RP = 1.16, IC (95 %) = [1.04; 1.29], *p* = 0.008); RP = 1.32, 95 % IC = [1.07; 1.62], *p* = 0.008); e (RP = 1.65, 95 % IC = [1.29; 2.10] *p* < 0.001), respectively, compared to the pre-surgery period (Supplementary Material – Table S2).

Table 3 shows the results of multiple linear regression models for the lifetime PD and weight change of each participant. Overall, the presence of psychiatric conditions at T0 and/or T1 did not show any association with %EWL. A marginal effect of time elapsed after MBS on %EWL was observed, with p-values of approximately 0.06. The only comparisons that yielded statistically significant results were those between the 72‒96 months and ≥ 96-months categories with the ≤ 24-months category. Patients who underwent MBS 72‒96 months ago gained about 12 % more excess weight than patients who had the surgery two years or less ago

**Table 3:** Multiple regression models of the association between %EWL and the presence of psychiatric disorder in T_0_ and/or T_1_ controlling variables sex, age, BMI, and time post-surgery^a^ for each disorder.

	Any Psychiatric Disorders	Any Bipolar Disorders	Any Mood Disorders	Any Substance Disorders	Any Anxiety Disorders	Any Eating Disorders
	β (SE)	β (SE)	β (SE)	β (SE)	β (SE)	β (SE)
**Intercept**	61.33 (12.73)	75.63 (9.56)	70.39 (10.89)	76.17 (9.15)	73.99 (10.22)	72.00 (9.93)
**Gender** (reference Women)						
**Men**	−6.71 (4.40)	−6.56 (4.43)	−6.39 (4.44)	−5.03 (4.59)	−6.62 (4.45)	−6.62 (4.43)
**Age** (mean 52 years)	−0.06 (0.16)	−0.08 (0.16)	−0.06 (0.16)	−0.10 (0.16)	−0.07 (0.16)	−0.06 (0.16)
**Time Post-Surgery** (reference ≤ 24 months)						
**24‒48 months**	−4.15 (5.79)	−4.67 (5.82)	−4.27 (5.85)	−3.55 (5.86)	−4.67 (5.86)	−4.15 (5.91)
**48‒72 months**	−1.35 (5.37)	−2.60 (5.33)	−2.36 (5.34)	−2.14 (5.32)	−2.56 (5.43)	−2.16 (5.40)
**72‒96 months**	**−12.61 (5.04)**	**−12.43 (5.08)**	**−12.63 (5.07)**	**−12.01 (5.07)**	**−12.52 (5.13)**	**−12.49 (5.07)**
**> 96 months**	−10.69 (5.45)	**−11.71 (5.46)**	**−11.54 (5.45)**	**−11.02 (5.44)**	**−11.51 (5.60)**	−10.93 (5.57)
**PD (T_0_/T_1_)** (reference not)						
**Yes**	12.01 (8.47)	−1.72 (3.56)	3.52 (5.73)	−4.72 (3.83)	0.12 (4.25)	1.90 (3.67)

a Values in bold were statistically significant at the 5 % confidence level compared to the reference category. Unstandardized coefficients: beta (β) and Standard error (SE); mean Body Mass Index in Day of Surgery (BMI DOS). Presence/absence of each psychiatric disorder in T_0_ and/or T_1_ (PD [T_0_/T_1_]).

The variation of %EWL based on TPS demonstrated that, although %EWL was consistently positive throughout all TPS periods, there were some notable changes over time. The average %EWL for TPS ≤ 24-months was 69.1 (SD = 22.1) and remained consistent for those who underwent MBS until 72-months. The mean %EWL decreased by 12 % for those who underwent surgery >72-months ago, with mean %EWL of 56.7 (SD = 19.2) and 57.9 (SD = 22.2) for TPS of 72‒96 months and > 96-months, respectively (Supplementary Material – Table S3).

## Discussion

This study addresses a significant gap in the existing research literature, as few studies have explored the long-term evolution of PDs and their relationship with weight changes following MBS. The results demonstrate that PDs can be associated over time, regardless of the time elapsed since undergoing surgery. There was a significant increase in lifetime mood disorders, bipolar disorder, and eating disorders. The frequency of longitudinal outcomes varied at different post-surgery time points. Depression and anxiety disorders were prevalent at all time points post-surgery. While anxiety disorders persisted throughout each TPS period, the prevalence of mood disorders varied across different TPS periods. The presence of PD did not show any association with %EWL. Continuous monitoring and support for bariatric patients is necessary due to variations throughout various post-surgery time intervals.

According to the literature, there is a decrease in the frequency of PD three years after undergoing MBS.^11^ However, these findings suggest that the amelioration of PDs might not be sustainable over time. The initial improvement of PD, when observed years after surgery, often diminishes with patients returning to preoperative levels of illness.^6^^,^^27^ Consistent with the existing literature, the present results indicate that PD can often occur after surgery.^8^^,^^10^

In neuropsychiatry, obesity is often associated with mood disorders, anxiety, and binge eating.^14^ Over the last few decades, there has been significant progress in understanding the complex course of many PDs. In this study, the authors found that these PDs varied in presentation. As anticipated in clinical settings, the results showed that patients who have undergone MBS had a higher incidence of mood disorders, particularly bipolar disorder, which may have been present in a subthreshold form prior to surgery and become more apparent afterward.^28^^,^^29^ Although MBS initially improves depressive symptoms, there is concern about potential relapse.^8^, ^9^, ^10^ Eating disorders tend to increase after 72-months and anxiety disorders were prevalent throughout all post-surgery periods. A longitudinal study has demonstrated that anxiety disorders significantly affect depression and binge eating.^30^ Further research is necessary to elucidate the intricate relationship between these mental conditions, particularly with regard to their longitudinal and temporal connections.

MBS can lead to significant weight loss but recurrent weight gain is not uncommon over the long term.^11^^,^^23^ Suboptimal clinical response has multifactorial causes, with some data suggesting an association with psychopathology, possibly related to challenges in coping with new social demands following weight loss.^6^^,^^27^^,^^31^ The authors found no association between PD and weight maintenance.

The link between obesity and neuropsychiatric syndromes implies a shared psychopathological basis, suggesting that maladaptive eating behaviors may contribute to long-term obesity.^14^ This study examined variables in the context of temporal variation in PD, with the objective of contributing to the ongoing debate about the potential influence of PD on clinical response after MBS. There is a complex interplay between these two clinical conditions (PD and obesity), which significantly affects global public health systems. PDs are characterized by progressive symptoms of mental illness that vary in intensity and manifestation and may be influenced by social and subjective contexts.^15^ In addition, while MBS can lead to substantial weight loss, a long-term clinical response is not always guaranteed due to the multifactorial nature of the disease.^11^^,^^24^^,^^31^ Long-term outcomes suggest that psychiatric comorbidities are influenced by specific characteristics present at various postoperative intervals.^32^^,^^33^ Although PD is prevalent after MBS, most patients experience improvements in postoperative quality of life.^34^ Therefore, optimal bariatric programs should include comprehensive behavioral health care, including regular psychological assessments, counseling, and interventions related to weight loss, spanning the preoperative phase to the long-term postoperative period.

The relationship between psychiatric disorders and weight loss patterns is not yet fully understood. Long-term research with patients who have undergone MBS presents a number of significant challenges, primarily due to the low adherence of some patients to the post-surgical treatment regimens proposed by surgical centers. This lack of adherence makes the tracking and analysis of long-term outcomes difficult. In this context, the present study was particularly challenging but also of great significance. The authors were able to obtain a representative sample of patients even nine years after surgery, which enabled us to comprehensively map psychiatric conditions and weight changes across different post-surgical periods. The potential contribution of this data is substantial. From a clinical perspective, the data provides valuable insights into the long-term psychiatric and weight-related outcomes of MBS, which can inform the development of more effective post-operative care strategies. From a research perspective, the findings can inform the design of future studies by highlighting key areas of focus and potential challenges in the long-term follow-up of MBS patients. Ultimately, this data can contribute to the improvement of treatment protocols and the attainment of better long-term outcomes for these patients. Further research is needed to explore this complex association and its long-term consequences.

### Limitations

The study has two potential bias issues which are important to note when interpreting the results. Firstly, there could be participant selection bias in the second wave, as patients with more mental disorders may have self-selected to participate. Secondly, although the study was conducted longitudinally, it was not a follow-up study. The data collection process occurred in two distinct waves, with a period of approximately nine years between assessments. During this time, there was no contact or direct interaction with the participating patients. Furthermore, the time gap between data collection points may have resulted in memory bias regarding specific symptoms. Participants may have inaccurate retrospective recall, remembering the past as better or worse than it was, which could have led to inaccuracies in their responses.

## Conclusion

The present study indicates that PDs exhibit a long-term association with MBS, irrespective of the time elapsed since surgery. However, it does not suggest a significant impact on weight loss maintenance. This article provides insights into the relationships between MBS and the temporal variation of PDs. Further investigations with an extended time frame are necessary to understand the complex interplay between these two clinical conditions, PD and obesity.

## Authors’ contributions

Leorides Severo Duarte-Guerra: Declares that have participated in the study and my specific contribution as follows: conception and design of the research, acquisition of data, analysis and interpretation of data, statistical analysis, drafting of the manuscript, critical revision of the manuscript for the important intellectual content.

Julia Faria Villares: Declares that have participated in the study and my specific contributions were as follows: conception and design of the research, participation in data collection as an interviewer, and preparation of the database and drafting of the manuscript.

Marco Aurélio Santo: Declares that have participated in the study and my specific contribution as follows: conception and design of the research, acquisition of data, and critical revision of the manuscript for the important intellectual content.

Francisco Lotufo Neto: Declares that have participated in the study and my specific contributions were as follows: conception and design of the research, analysis, and interpretation of data, drafting of the manuscript, critical revision of the manuscript for the important intellectual content.

Yuan Pang Wang: Declares that have participated in the study and my specific contribution as follows: conception and design of the research, acquisition of data, analysis, and interpretation of data, statistical analysis, drafting of the manuscript, critical revision of the manuscript for the important intellectual content, and supervision as the principal investigator.

## Conflicts of interest

The authors declare no conflicts of interest.

## Appendix Supplementary materials

Image, application 1
