# Supplementary material for: Relationship between psychiatric disorders and loss weight among patients underwent metabolic and bariatric surgery: A reassessment observational study after nine years
Source: Clinics (Sao Paulo). 2024 Oct 20;79:100517. doi: 10.1016/j.clinsp.2024.100517 (PMC11533480; doi:10.1016/j.clinsp.2024.100517)
Supplement: Supplementary file 1 [file mmc1.docx]

**CLINICS-D-24-00531_Supplementary Material**

**Supplementary Material Table S1** Socio-demographic characteristics of patients dropped out (T_0_-2011) and reassessed (T_1_-2019/2020).

|  | **Follow up in 2019/2020** | | **Total (n = 346)** | **p** |
| --- | --- | --- | --- | --- |
|  | **No (n = 204)** | **Yes (n = 142)** |  |  |
| **Sex (n, %)** |  |  |  |  |
| Women | 159 (77.9) | 117 (82.4) | 276 (79.8) | 0.31 |
| Men | 45 (22.1) | 25 (17.6) | 70 (20.2) |  |
| **Age (years)** |  |  |  |  |
| Mean (sd) | 42 (12) | 44 (11) | 43 (11) | 0.07 |
| Median (1°‒3° quartile) | 41 (33‒50) | 45 (36‒52.75) | 43 (35-51) |  |
| **Marital Status (n, %)** |  |  |  |  |
| Married | 100 (49) | 79 (55.6) | 179 (51.7) | 0.27 |
| Widow | 16 (7.8) | 8 (5.6) | 24 (6.9) |  |
| Separated | 32 (15.7) | 27 (19) | 59 (17.1) |  |
| Single | 56 (27.5) | 28 (19.7) | 84 (24.3) |  |
| **Education (n, %)** |  |  |  |  |
| 1‒8 years | 76 (37.3) | 48 (33.8) | 124 (35.8) | 0.80 |
| 9‒12 years | 89 (43.6) | 65 (45.8) | 154 (44.5) |  |
| >12 years | 39 (19.1) | 29 (20.4) | 68 (19.7) |  |
| **Employed (n, %)** |  |  |  |  |
| No | 79 (38.7) | 55 (38.7) | 134 (38.7) | 0.99 |
| Yes | 125 (61.3) | 87 (61.3) | 212 (61.3) |  |
| **Tobacco use (n, %)** |  |  |  |  |
| Never smoker | 114 (55.9) | 80 (56.3) | 194 (56.1) | 0.46 |
| Current smoker | 27 (13.2) | 13 (9.2) | 40 (11.6) |  |
| Ex-smoker | 63 (30.9) | 49 (34.5) | 112 (32.4) |  |
| **BMI (kg/m^2^)** |  |  |  |  |
| Mean (sd) | 47.98 (8.19) | 46.45 (6.33) | 47.35 (7.51) | 0.20 |
| Median (1°‒3° quartile) | 46.77 (42.55‒52.2) | 46.42 (41.77‒50.48) | 46.66 (42.34‒51.12) |  |
| **GAF** |  |  |  |  |
| Mean (sd) | 76.85 (12.35) | 76.19 (12.32) | 76.58 (12.32) | 0.59 |
| median (1°‒3° quartile) | 80 (70‒90) | 79 (70‒87.75) | 80 (70‒90) |  |
| **Number of disorders – lifetime (n, %)** |  |  |  |  |
| 0 | 40 (19.6) | 25 (17.6) | 65 (18.8) | 0.92 |
| 1 | 42 (20.6) | 27 (19) | 69 (19.9) |  |
| 2 | 45 (22.1) | 34 (23.9) | 79 (22.8) |  |
| 3+ | 77 (37.7) | 56 (39.4) | 133 (38.4) |  |
| **Number of disorders – current (n, %)** |  |  |  |  |
| 0 | 82 (40.2) | 58 (40.8) | 140 (40.5) | 0.89 |
| 1 | 57 (27.9) | 42 (29.6) | 99 (28.6) |  |
| 2 | 30 (14.7) | 22 (15.5) | 52 (15) |  |
| 3+ | 35 (17.2) | 20 (14.1) | 55 (15.9) |  |

SD, Standard Deviation; BMI, Body Mass Index; GAF, Global Assessment of Function.

**Supplementary Material Table S2** Estimated Prevalence and 95% CI for Psychiatric Disorders according to the time elapsed after bariatric surgery.

| **TPS** | **Evaluation** | **Any Mood Disorders** | **Any Bipolar Disorders** | **Any Substance Disorders** | **Any Anxiety Disorders** | **Any Eating Disorders** |
| --- | --- | --- | --- | --- | --- | --- |
| ≥ 24 months | T_0_ | 0.74 [0.63; 0.87] | 0.44 [0.31; 0.62] | 0.12 [0.05; 0.27] | 0.60 [0.50; 0.71] | 0.41 [0.30; 0.55] |
|  | T_1_ | 0.86 [0.74; 1.00] | 0.58 [0.43; 0.80] | 0.17 [0.08; 0.36] | 0.64 [0.53; 0.77] | 0.67 [0.53; 0.86] |
| 24‒48 months | T_0_ | 0.61 [0.47; 0.79] | 0.39 [0.28; 0.56] | 0.24 [0.14; 0.43] | 0.60 [0.46; 0.79] | 0.25 [0.15; 0.40] |
|  | T_1_ | 0.71 [0.55; 0.90] | 0.52 [0.37; 0.73] | 0.35 [0.20; 0.60] | 0.65 [0.50; 0.84] | 0.41 [0.26; 0.64] |
| 48‒72 months | T_0_ | 0.71 [0.59; 0.85] | 0.42 [0.30; 0.58] | 0.18 [0.09; 0.35] | 0.55 [0.42; 0.73] | 0.32 [0.22; 0.47] |
|  | T_1_ | 0.82 [0.69; 0.97] | 0.55 [0.40; 0.75] | 0.26 [0.14; 0.47] | 0.59 [0.45; 0.78] | 0.53 [0.38; 0.74] |
| < 72‒96 months | T_0_ | 0.77 [0.67; 0.88] | 0.46 [0.35; 0.60] | 0.16 [0.09; 0.28] | 0.59 [0.48; 0.74] | 0.41 [0.31; 0.55] |
|  | T_1_ | 0.89 [0.80; 0.99] | 0.60 [0.47; 0.77] | 0.23 [0.13; 0.39] | 0.64 [0.52; 0.79] | 0.68 [0.55; 0.84] |
| > 96 months | T_0_ | 0.64 [0.54; 0.77] | 0.32 [0.21; 0.48] | 0.16 [0.08; 0.32] | 0.52 [0.38; 0.71] | 0.23 [0.15; 0.37] |
|  | T_1_ | 0.75 [0.63; 0.88] | 0.42 [0.28; 0.62] | 0.23 [0.12; 0.43] | 0.56 [0.41; 0.76] | 0.39 [0.25; 0.59] |

TPS, Time Post-Surgery.

**Supplementary Material Table S3** Percentage of excess weight loss (%EWL) by Time Post-Surgery (TPS).

| **TPS (months)** | **Mean (SD)** | **Median (25^th^–75^th^ percentile)** |
| --- | --- | --- |
| **≤ 24** | 69.1 (22.1) | 68.7 (54.4‒78.7) |
| **24‒48** | 64.8 (17.9) | 68.6 (50.0‒75.4) |
| **48‒72** | 66.6 (17.7) | 66.0 (56.8‒76.1) |
| **72‒96** | 56.7 (19.2) | 58.6 (44.6‒67.2) |
| **> 96** | 57.9 (22.2) | 58.8 (46.0‒79.0) |

SD, Standard Deviation.
